# Structural and Functional Changes in Subcortical Vascular Mild Cognitive Impairment: A Combined Voxel-Based Morphometry and Resting-State fMRI Study

**DOI:** 10.1371/journal.pone.0044758

**Published:** 2012-09-19

**Authors:** Liye Yi, Jinhui Wang, Longfei Jia, Zhilian Zhao, Jie Lu, Kuncheng Li, Jianping Jia, Yong He, Chuanlu Jiang, Ying Han

**Affiliations:** 1 Department of Neurology, Xuanwu Hospital, Capital Medical University, Beijing, China; 2 The Second Affiliated Hospital, Harbin Medical University, Harbin, China; 3 State Key Laboratory of Cognitive Neuroscience and Learning, Beijing Normal University, Beijing, China; 4 Department of Radiology, Xuanwu Hospital, Capital Medical University, Beijing, China; 5 Department of Neurology, Beijing Tongren Hospital, Capital Medical University, Beijing, China; Hangzhou Normal University, China

## Abstract

The present study aimed to investigate changes in structural gray matter (GM) volume and functional amplitude of spontaneous low-frequency oscillations (LFO) and functional connectivity density in patients with subcortical vascular mild cognitive impairment (svMCI). Structural MRI and resting-sate functional MRI data were collected from 26 svMCI patients and 28 age- and gender-matched healthy controls. Structurally, widespread GM atrophy was found in the svMCI patients that resided primarily in frontal (e.g., the superior and middle frontal gyri and medial prefrontal cortex) and temporal (the superior and inferior temporal gyri) brain regions as well as several subcortical brain sites (e.g., the thalamus and the caudate). Functionally, svMCI-related changes were predominantly found in the default mode network (DMN). Compared with the healthy controls, the svMCI patients exhibited decreased LFO amplitudes in the anterior part of the DMN (e.g., the medial prefrontal cortex), whereas increased LFO amplitudes in the posterior part of the DMN (e.g., the posterior cingulate/precuneus). As for functional connectivity density, the DMN regions (e.g., the posterior cingulate/precuneus, the medial prefrontal cortex and the middle temporal gyrus) consistently exhibited decreased functional connectivity. Finally, the overall patterns of functional alterations in LFO amplitudes and functional connectivity density remained little changed after controlling for structural GM volume losses, which suggests that functional abnormalities can be only partly explained by morphological GM volume changes. Together, our results indicate that svMCI patients exhibit widespread abnormalities in both structural GM volume and functional intrinsic brain activity, which have important implications in understanding the pathophysiological mechanism of svMCI.

## Introduction

Mild cognitive impairment (MCI) refers to a transitional state between normal aging and dementia and is characterized by various symptom clusters. As a clinically heterogeneous syndrome, MCI can be classified into subtypes of amnestic and non-amnestic phenotypes, both of which can be further subdivided into two groups that exhibit cognitive deficits in single and multiple functional domains [1]–[4]. Numerous previous studies have primarily focused on amnestic MCI (aMCI), due to the relatively high incidence and straightforward clinical diagnosis of this type of MCI [5]–[8]. In recent years, the non-amnestic MCI, particularly the subcortical vascular MCI (svMCI) subtype, has begun to attract attention [9]–[12]. Also, svMCI has been proposed as a prodromal stage of subcortical vascular dementia (SVaD) [11], [13], [14], which is one of the most common types of vascular dementia [15], [16]. Recent evidence suggests that svMCI is potentially reversible [17], [18]; therefore, characterizing the structural and functional brain abnormalities in the atypical population with svMCI can provide insights into understanding the pathophysiological mechanism of svMCI.

Previous studies have shown that human brain structure and function can be explored *in vivo* by neuroimaging techniques, such as structural MRI (sMRI) and resting-state functional MRI (R-fMRI). Importantly, sMRI provides rich morphological information of brain tissues and has been extensively used to reveal cortical atrophy, thinning and deformation in response to pathological attacks. With respect to svMCI, Seo et al. [11] investigated the cortical thickness of the brains of patients with svMCI and found significant cortical thinning in the inferior frontal and orbitofrontal gyri, the anterior cingulate, the insula, the superior temporal gyrus and the lingual gyrus in the patients. Despite the importance of the insights into svMCI that were revealed by cortical thickness analysis, this approach cannot deal with subcortical regions (e.g., the thalamus and the caudate). Subcortical gray matter (GM) atrophy has been reported in patients with subcortical ischemic vascular dementia, which is a clinical state proposed as the advanced stage of svMCI [19]. This finding suggests the implications of subcortical brain sites in subcortical vascular etiology. Thus, identifying the GM atrophy pattern over the whole brain in svMCI patients is clearly warranted but currently is largely unknown.

Recently, R-fMRI has emerged as an effective, noninvasive imaging technique that is used to study the intrinsic functional architecture of the human brain when subjects are not engaged in external tasks. R-fMRI measures the spontaneous neural activity as low-frequency (typically <0.1 Hz) oscillations (LFO) of BOLD signals that are crucial for the understanding of the intrinsic functional organization of the human brain [20], [21]. Moreover, R-fMRI provides a promising avenue for exploring changes in the spontaneous neural activity that are associated with various brain disorders [22], [23]. One previous study that utilized position emission tomography reported svMCI-related hypometabolism in multiple brain regions, such as the inferior frontal gyrus, the thalamus and the caudate [10]. However, to the best of our knowledge, there are notably few studies that have investigated the intrinsic functional architecture of svMCI patients by R-fMRI.

In the current study, we performed a multimodal imaging analysis by combining sMRI and R-fMRI to explore both structural and functional brain abnormalities in patients with svMCI. Functionally, we utilized two test-retest reliable measures, the amplitude of low-frequency fluctuation (ALFF) [24], [25] and functional connectivity density [26] to characterize spontaneous LFO from two perspectives of local fluctuation power and interregional connectivity, respectively. LFO amplitude reflects the intensity of the regional spontaneous brain activity [27] and functional connectivity density quantifies the connectivity density of a region across the whole brain. Structurally, we used a voxel-based morphometry (VBM) approach [28], [29] to perform an unbiased whole-brain analysis of GM volume. Additionally, we also tested whether and to what extent the functional abnormalities in the svMCI patients could be influenced by structural changes.

## Materials and Methods

### Participants

Fifty-four right-handed participants, including 26 svMCI patients (11 male, ages 46–81 years) and 28 healthy controls (HC) (12 male, ages 50–79 years) participated in this study. The recruited patients were outpatients who were registered at the neurology department of Xuanwu Hospital, Capital Medical University, Beijing, China. The healthy controls were community residents from Beijing who were recruited by advertisements. The diagnosis of svMCI was performed by two experienced neurologists in consensus according to criteria [2], [30]–[32] that included the following: 1) subjective cognitive complaints reported by the participant or his/her caregiver; 2) objective cognitive impairments, although not meeting the Diagnostic and Statistical Manual of Mental Disorders, fourth edition (DSM-IV) criteria for dementia; 3) normal or near-normal performance of general cognitive functioning and no or minimum impairments of daily life activities; 4) a Clinical Dementia Rating Scale (CDR) score = 0.5; 5) a Mini-Mental State Examination (MMSE) score ≥24; and 6) subcortical vascular causes of the cognitive impairments according to a) moderate to severe white matter (WM) hyperintensity in at least one region with a Wahlund rating scale score ≥2 (Wahlund et al., 2001) and/or multiple lacunar infarcts in the periventricular and deep WM structures (Wahlund rating scale score ≥2; diameter <15 mm) on T2-weighted or FLAIR images, and b) evident neurological signs of hemiparesis, lower facial weakness, Babinski sign, dysarthria, sensory deficit, gait disorder, urgent urination or motor slowness that were assessed by general and neurological examination or reported by the participant or his/her caregiver. The exclusive criteria for svMCI included [32], [33]: 1) deficits in memory and other cognitive functions in the absence of focal lesionson brain imaging; 2) cognitive impairments as a result of other causes, such as tumor, epilepsy, traumatic brain injury, multiple sclerosis, psychiatric disease, systemic disease (e.g., thyroid dysfunction, severe anemia, syphilis and HIV), alcohol or drug abuse; 3) a suffering of visual abnormalities, severe aphasia or palsy that made clinical assessments infeasible; 4) signs of large vessel disease, such as cortical and/or corticosubcortical non-lacunar territorial infarcts and watershed infarcts or hemorrhages; and 5) diseases that led to white matter lesions, such as normal pressure hydrocephalus, multiple sclerosis, sarcoidosis or brain irradiation. All healthy controls had no history of any neurological or psychiatric disorders, no cognitive complaints and no abnormalities in their conventional brain MRI images. All of the participants underwent a standardized clinical evaluation protocol, which included a general and neurological examination, a global cognitive level test (i.e., CDR and MMSE) and other cognitive assessments[i.e., Activities of Daily Living (ADL) and Auditory-Verbal Learning Test (AVLT)]. Table 1 presents the details of the demographics and clinical characteristics of all the participants. This study was approved by the medical research ethics committee and institutional review board of Xuanwu Hospital, Capital Medical University, Beijing, China, and written informed consent was obtained from each participant.

**Table 1:** Demographics and clinical characteristics of the participants.

	HC (n = 28)	svMCI (n = 26)	P-value
Gender (male/female)	12/16	11/15	0.967^a^
Age (years)	50–79 (65.3±8.1)	46–81 (66.7±9.5)	0.549^b^
Education (years)	0–22 (12.3±5.0)	0–18 (9.9±4.4)	0.066^b^
MMSE	26–30 (29.1±1.2)	21–30 (25.7±2.7)	<10^−6b^
AVLT-immediate recall	6.3–14.7 (9.4±2.2)	3.7–9.7 (6.3±1.8)	<10^−5b^
AVLT-delayed recall	7–15 (10.8±2.7)	1–12 (6.3±3.1)	<10^−6b^
AVLT-recognition	9–15 (12.8±1.7)	4–14 (10.0±3.0)	<10^−3b^

Data are presented as the range of min – max (mean ± SD). HC, healthy controls; svMCI, subcortical vascular mild cognitive impairment; MMSE, Mini-Mental State Examination; AVLT, Auditory Verbal Learning Test.

a The P value was obtained by a two-tail Pearson chi-square test.

b The P value was obtained by a two-sample two-tail t-test.

### Data acquisition

All images were acquired using a 3.0 T Siemens scanner at Xuanwu Hospital, Capital Medical University. During the scan, foam pads and headphones were used to reduce head motion and scanner noise as much as possible. Structural images were collected using a sagittal magnetization-prepared rapid gradient echo (MP-RAGE) three-dimensional T1-weighted sequence [repetition time (TR) = 1900 ms; echo time (TE) = 2.2 ms; inversion time (TI) = 900 ms; flip angle (FA) = 9°; number of slices = 176; slice thickness = 1.0 mm; data matrix = 256×256; field of view (FOV) = 256×256 mm^2^]. Resting-state functional images were acquired using an echo-planar imaging (EPI) sequence [TR = 2000 ms; TE = 40 ms; FA = 90°; number of slices = 28; slice thickness = 4 mm; gap = 1 mm; data matrix = 64×64; FOV = 256×256 mm^2^]. The subjects were instructed to lie quietly in the scanner with their eyes closed and to remain stable as much as possible during the data acquisition. The functional scan lasted for 478 s (239 volumes) in total.

### Structural image analysis

To identify brain regions that showed morphological GM volume changes in the svMCI patients relative to healthy controls, we performed a VBM analysis on the structural images using the SPM8 package (http://www.fil.ion.ucl.ac.uk/spm). Briefly, individual structural images (3D T1-weighted anatomical images) were co-registered to their corresponding mean functional images (after time and head motion correction; see below for the details) using a linear transformation [34]. The transformed structural images were subsequently segmented into GM, WM and cerebrospinal fluid (CSF) using a new segment procedure provided in the SPM8 toolbox. Then DARTEL toolbox was used to enhance inter-subject registration of the resultant GM, WM and CSF maps [35]. After inter-subject coregistration, all the GM, WM and CSF maps were further transformed into the Montreal Neurological Institute (MNI) space, modulated to compensate for spatial normalization effects and spatially smoothed using a 6-mm full width at half maximum (FWHM) Gaussian kernel. These smoothed images were used to identify the brain regions that exhibited GM changes in the svMCI patients. Notably, between-group comparisons were restricted within a GM mask, which was derived by thresholding (cut-off = 0.25) the mean GM map of all participants (images before modulation and after normalization).

### Functional image analysis

#### Image preprocessing

Data preprocessing of the functional images was also conducted using the SPM5 package (http://www.fil.ion.ucl.ac.uk/spm). Before the preprocessing, the first five volumes of each subject were discarded to account for the effects of T1 equilibration and the subjects' adaptation to the circumstances, leaving 234 images for further analysis. The remaining functional images were corrected for intra-volume acquisition time delays between slices and for inter-volume geometrical displacements due to head movement. No participants were excluded according to the criteria of a displacement larger than 3 mm or an angular rotation greater than 3° in any of the x, y or z directions throughout the course of scan. There was no significant difference in the mean absolute values of head motion in any direction (all P>0.05). Next, all functional data were normalized to the MNI space by applying transformation parameters that were obtained in the segmentation of the structural images to those time- and motion-corrected functional images. The resultant normalized functional images underwent further spatial smoothing (6-mm FWHM Gaussian kernel) and a removal of linear trends. Finally, temporal band-filtering (0.01 Hz<f<0.1 Hz) was performed to reduce the effects of low-frequency drifts and high frequency noise [36], [37]. To reduce non-neural variance in the time series of each voxel, several confounding factors were regressed out including WM signals, CSF signals and six head motion profiles.

#### ALFF calculation

ALFF was calculated using REST software (http://www.restfmri.net) [38]. Briefly, for a given voxel, the time series was first converted to the frequency domain using a fast Fourier transform. Next, the square root of the power spectrum was computed and averaged across 0.01–0.1 Hz. The averaged square root was termed ALFF at the given voxel [24]. To account for the global effects of variability across participants, individual ALFF at each voxel was divided by the mean ALFF value within the brain GM mask that was generated above.

Functional connectivity densityIn addition to ALFF, we also calculated voxel-wise functional connectivity density. For a given voxel, the time series was correlated with all the other voxels in the GM brain mask. The functional connectivity density of the given voxel is then computed as the number of voxels that showed high correlations (>0.3) with the given voxel. Given the ambiguous interpretation of negative correlations, only positive correlations were conservatively considered in the current study.

### Statistical analysis

#### Within-group ALFF analysis

To explore within-group distributions of ALFF, one-sample one-tailed t-tests were performed for each group to determine the brain regions showing larger-than-mean ALFF (i.e., reference value = 1 for t-tests). The possible effects of age, gender and education level were controlled. The statistical threshold for within-group analysis was set as P<0.01 (corrected) by combining height threshold P<0.001 for individual voxels and extent threshold P<0.01 (i.e., cluster size >324 mm^3^) as determined by Monte Carlo simulations [39].

#### Between-group comparisons in GM volume, ALFF and functional connectivity density

To determine between-group differences in GM volume, ALFF and functional connectivity density, multiple linear regression analyses were performed in a voxel-wise manner (dependent variable: GM volume, ALFF or functional connectivity density; independent variable: group indicator variable with 1 indicating clinical and 0 healthy states) with age, gender and education as common unconcerned covariates. Specifically, total intracranial volume (sum of GM, WM and CSF maps) was added as extra covariate for GM volume between-group comparison and maximum and root mean squares of head motion (translation and rotation) for ALFF and functional connectivity density between-group comparisons. Furthermore, to determine the contribution of structural changes on functional abnormalities, between-group comparisons of ALFF and functional connectivity density were also performed, with structural GM volume serving as another extra-explanatory variable. This specific statistical analysis was performed using the REST software (http://www.restfmri.net) [38]. All results were presented at the statistical threshold of P<0.01 (corrected) by combined height threshold P<0.01 for individual voxels and extent threshold P<0.01 (i.e., cluster size >948 mm^3^ for functional results and cluster size >972 mm^3^ for structural results), as determined by Monte Carlo simulations [39].

#### Relationship of ALFF/functional connectivity density/GM volume with clinical variables

To examine the relation of imaging results (both structural and functional) with the cognitive performance of the patients (MMSE and AVLT), we performed multiple linear regression analyses that were restricted within the brain regions that showed significant between-group differences. Age, gender and education were also treated as unconcerned covariates in the model.

## Results

### Demographic and clinical characteristics of the participants

As shown in Table 1, there were no significant differences in age (P = 0.549), gender (P = 0.967) or years of education (P = 0.066) between the svMCI patients and the healthy controls. However, the svMCI patients showed significantly lower scores for the MMSE (P<10^−6^), the AVLT-immediate recall (P<10^−5^), the AVLT-delayed recall (P<10^−6^) and the AVLT-recognition (P<10^−3^) in comparison with the healthy controls.

### Differences in structural GM volume

**Figure 1:**
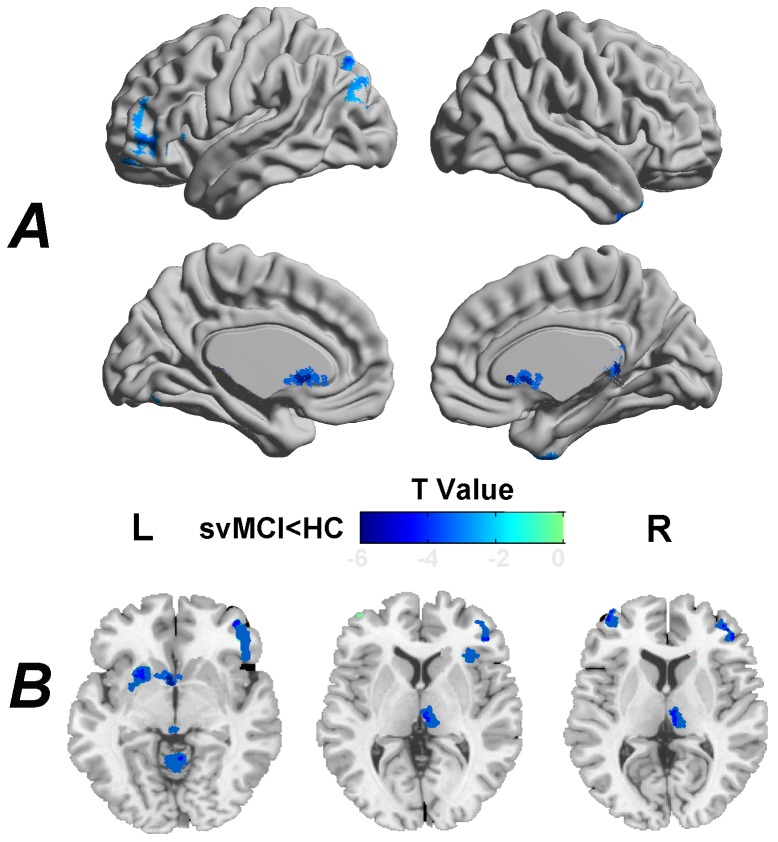
Cortical (A) and subcortical (B) GM volume loss in svMCI. The svMCI patients showed decreased GM volume in several frontal, temporal, occipital and subcortical brain regions. For the details, see Table 2. The statistical threshold was set at P<0.01 for individual voxels and cluster size >948 mm^3^, which corresponded to a corrected P<0.01 determined by Monte Carlo simulations. Cortical (A) t statistical maps were rendered by using the BrainNet Viewer (http://www.nitrc.org/projects/bnv/). Subcortical (B) t statistical map was overlaid on a single anatomical image (i.e., ch2bet.nii) provided in the MRIcroN software (http://www.cabiatl.com/mricro/). R, right; L, left.

The svMCI patients exhibited decreased GM volume not only in cortical (Fig. 1A and Table 2) but also in subcortical brain regions (Fig. 1B and Table 2). The cortical regions included several frontal (i.e., the bilateral SFG and the MFG), temporal (i.e., the right STG and the right ITG) and one occipital [i.e., the left middle occipital gyrus (MOG)] region and one parietal region (i.e., the IPL). The subcortical regions included the caudate (CAU) and the thalamus (THA). There were no brain regions that showed increased GM volume in the patients compared to that of the healthy controls.

**Table 2:** Regions showing GM volume atrophy in the svMCI patients.

			MNI coordinate (mm)			
Brain regions	BA	Vol (mm^3^)	X	Y	Z	Maximum T
Right STG/ITG	38	560	33	12	−40	−4.52
Left CAU	10	256	−18	4	27	−4.14
Right THA	23	351	−8	−12	6	−4.05
Left MOG/IPL	19/39	111	−31	−81	35	−4.01
Left SFG/MFG	11/47	300	−46	45	−1	−3.57

BA, Brodmann area; Vol, cluster volume; X, Y, Z, coordinates of peak locations; T, t statistical value at peak locations. STG, superior temporal gyrus; ITG, inferior temporal gyrus; CAU, caudate; THA, thalamus; MOG, middle occipital gyrus; IPL, inferior parietal lobule; SFG, superior frontal gyrus; MFG, middle frontal gyrus.

### Within-group ALFF and functional connectivity patterns

**Figure 2:**
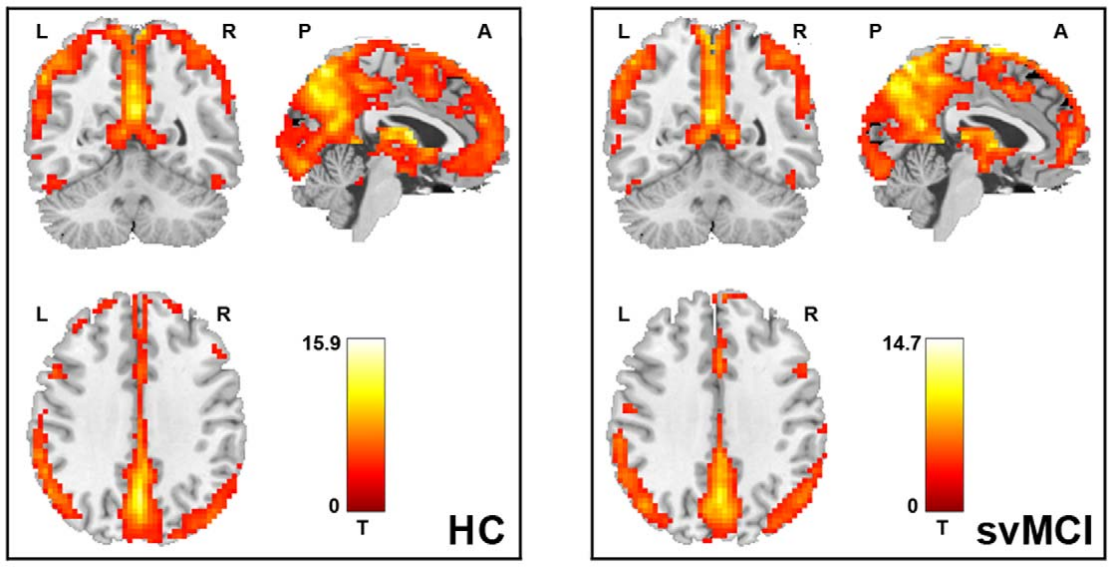
Within-group ALFF maps of the HC (left) and svMCI (right) groups. Widely spread regions of parietal, frontal, occipital and temporal lobes exhibited high ALFF values in the both groups. Note that the posterior cingulate (PCC) and adjacent precuneus (PCu) showed the highest ALFF values in the both groups. The statistical threshold was set at P<0.001 for individual voxels and cluster size >324 mm^3^, which corresponded to a corrected P<0.01 determined by Monte Carlo simulations. L, left; R, right; P, posterior; A, anterior.

**Figure 3:**
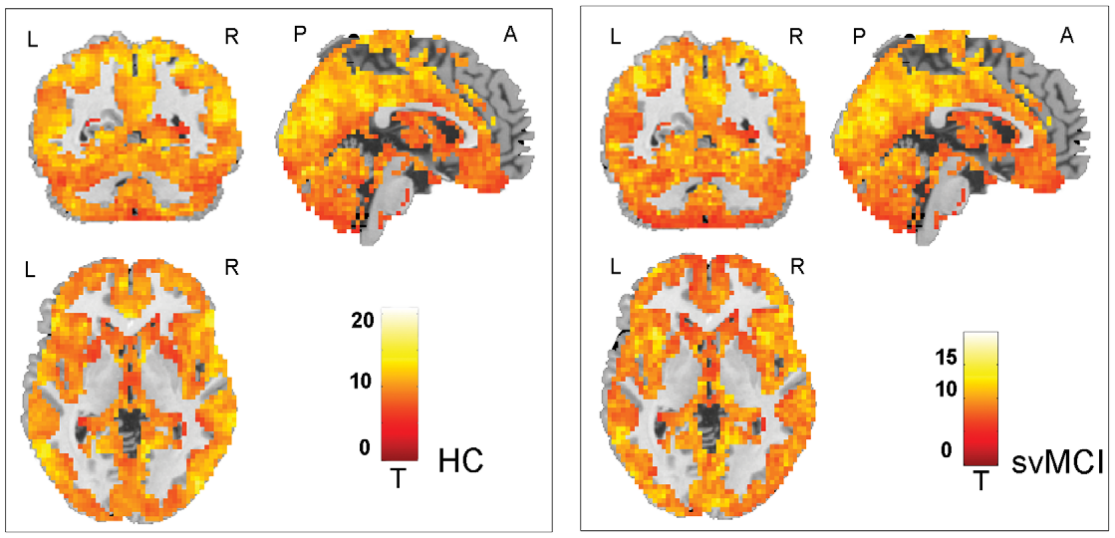
Within-group functional connectivity maps of the HC (left) and svMCI (right) groups. Widely spread regions of parietal, frontal, occipital and temporal lobes exhibited high functional connectivity values in the both groups. Note that the posterior cingulate (PCC) and adjacent precuneus (PCu) showed the highest functional connectivity density in the both groups. The statistical threshold was set at P<0.001 for individual voxels and cluster size >324 mm^3^, which corresponded to a corrected P<0.01 determined by Monte Carlo simulations. L, left; R, right; P, posterior; A, anterior.

Figure 2&3 shows the ALFF and functional connectivity maps of the HC and svMCI groups. Visual inspection indicated that widely spread regions exhibited high ALFF and functional connectivity values in the both groups, such as parietal regions of the posterior cingulate (PCC) and adjacent precuneus (PCu), postcentral gyrus and superior parietal lobule (SPL), frontal regions of the medial prefrontal cortex (MPFC), superior (SFG) and middle (MFG) frontal gyri, occipital regions of the lingual gyrus and calcarine, and temporal regions of the superior temporal gyrus and fusiform gyrus. Of note, the PCC/PCu had the highest ALFF values in the both groups, which are the key nodes of the default mode network (DMN) [40], [41]. We also noted that the amplitude of t statistic was higher in the HC group as compared with the svMCI group. Here these within-group maps were merely for visualizing ALFF and functional connectivity density.

### Differences in functional amplitudes of LFO

**Figure 4:**
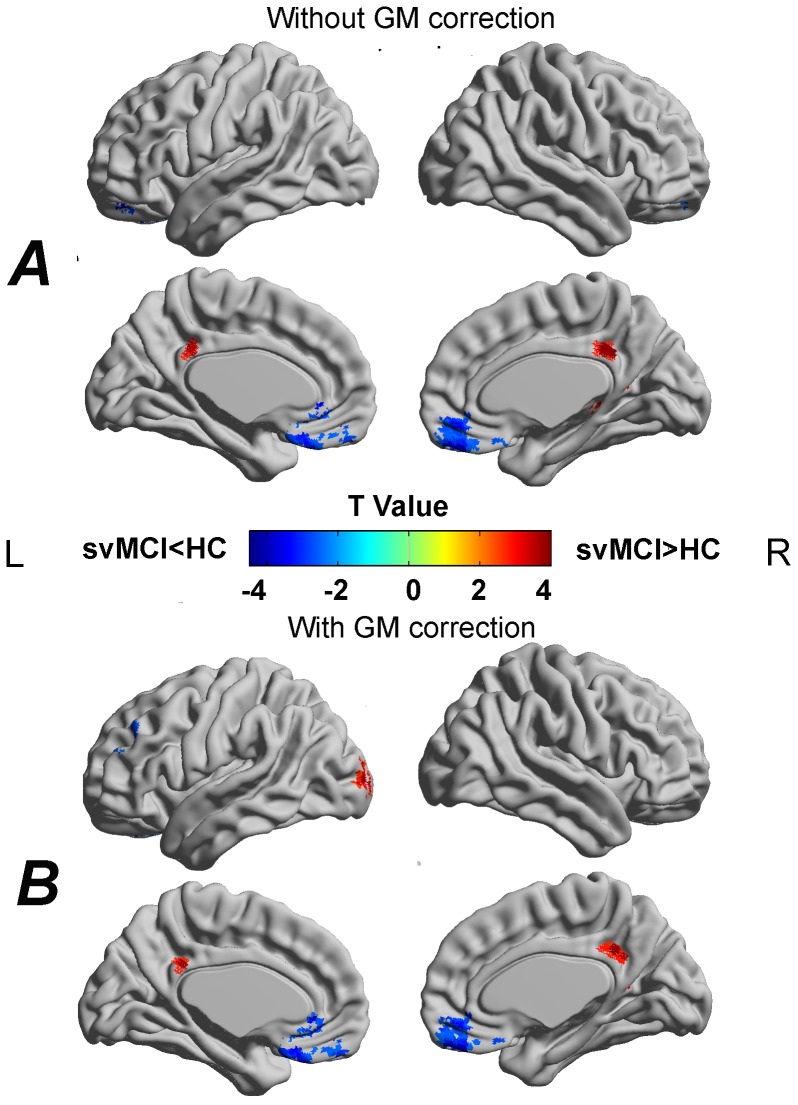
Between-group differences in the amplitude of LFO without (A) and with (B) correcting GM volume. The svMCI patients showed decreased LFO amplitudes in frontal and temporal regions whereas increased LFO amplitudes in parietal and occipital regions. For the details, see Table 3. Of note, the between-group differences in the LFO amplitudes exhibited highly similar patterns between with and without correcting GM volume. The statistical threshold was set at P<0.01 for individual voxels and cluster size >972 mm^3^, which corresponded to a corrected P<0.01 determined by Monte Carlo simulations. The t statistical maps were rendered by using the BrainNet Viewer (http://www.nitrc.org/projects/bnv/).R, right; L, left.

Compared with the HC, the svMCI patients showed significantly decreased ALFF predominantly in the anterior part of the DMN (i.e., the bilateral MPFC). The svMCI patients also showed increased ALFF in regions that primarily encompassed the posterior part of the DMN (i.e., the right PCC/PCu) (Fig. 4A and Table 3). In addition, the right hippocampus (HIP) and the right thalamus (THA) also showed increased ALFF in the svMCI patients.

**Table 3:** Regions showing differences in amplitudes of LFO between the svMCI patients and healthy controls.

			MNI coordinate (mm)			
Brain regions	BA	Vol (mm^3^)	X	Y	Z	Maximum T
*Decreased ALFF in the svMCI patients*						
Bilateral MPFC	11	7560	3	42	−9	−3.92
*Increased ALFF in the svMCI patients*						
Right PCC/PCu	31	1215	12	−54	15	3.76
Right HIP/THA	27	1350	24	−39	0	3.73

BA, Brodmann area; Vol, cluster volume; X, Y, Z, coordinates of peak locations; T, t statistical value at peak locations. MPFC, medial frontal cortex; PCC/PCu, posterior cingulate/precuneus; HIP, hippocampus; THA, thalamus.

### Differences in functional connectivity density

**Figure 5:**
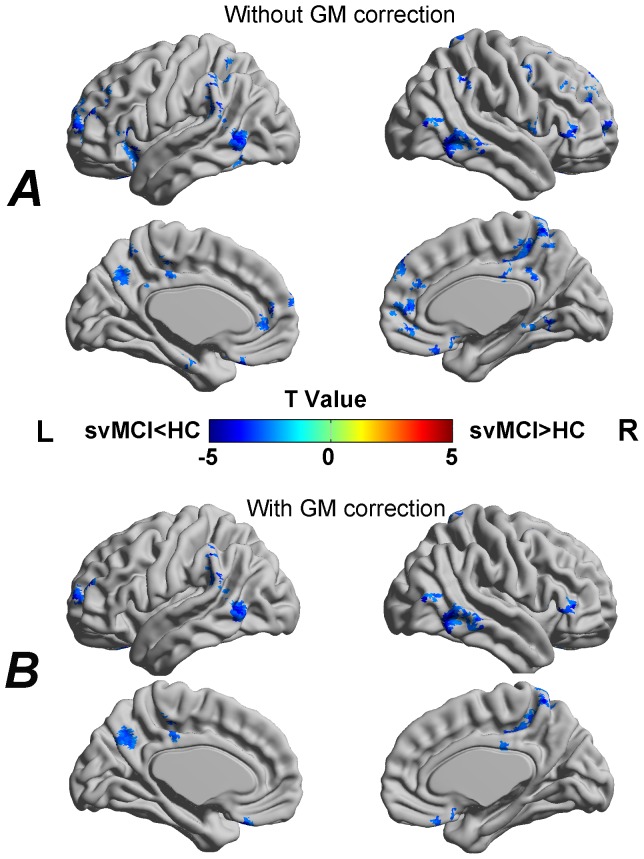
Between-group differences in functional connectivity density without (A) and with (B) correcting GM volume. The svMCI patients showed decreased functional connectivity density in frontal, temporal and parietal regions. Of note, several brain regions which showed functional connectivity density reduction were not significant after GM correction. The statistical threshold was set at P<0.01 for individual voxels and cluster size >972 mm^3^, which corresponded to a corrected P<0.01 determined by Monte Carlo simulations. The t statistical maps were rendered by using the BrainNet Viewer (http://www.nitrc.org/projects/bnv/).R, right; L, left.

Compared with the HC, the svMCI patients showed significantly decreased functional connectivity density mainly in the DMN regions, including the PCC/PCu, MPFC, SFG and inferior parietal lobule (IPL) and HIP (Fig. 5A). The inferior frontal gyrus (IFG) and MFG also showed decreased functional connectivity density in svMCI.

### Effects of structural GM atrophy on functional ALFF and functional connectivity density

Figure 4B shows the between-group differences in the amplitude of spontaneous LFO after controlling for GM volume. Figure 5B shows the between-group differences in functional connectivity density after controlling for GM volume. We found that although the amplitudes of t statistic and the cluster size decreased slightly, the ALFF and functional connectivity density changes exhibited highly similar patterns with those without correcting GM volume. This suggests that functional alterations observed in the svMCI patients can be only partly explained by structural GM atrophy.

### Relationship between imaging changes and clinical variables

No significant correlations were found for structural GM volume, functional LFO amplitude and functional connectivity density with any of the clinical variables, which included MMSE, AVLT-immediate recall, AVLT-delayed recall or AVLT-recognition in the svMCI patients.

## Discussion

In the present study, we investigated svMCI-related alterations in both functional spontaneous LFO and the structural GM volume by combining resting-state fMRI and structural MRI techniques. We found that compared with HC, the svMCI patients exhibited a functional decrease in the amplitude of spontaneous LFO, primarily in the anterior part of the DMN, whereas the amplitude was increased in the posterior part of the DMN. We also found decreased functional connectivity density in both anterior and posterior part of the DMN in svMCI patients. Structural GM atrophy was distributed over brain sites, predominantly the frontal lobe, the temporal lobe and the subcortex. These findings provide new insights into the neurophysiological alterations of the brain that are induced by svMCI.

### Decreased spontaneous LFO in anterior DMN in svMCI

We found that the svMCI patients exhibited decreased spontaneous LFO in the MPFC, which belongs to the DMN. The DMN is a set of brain regions that routinely exhibit activity decreases during attention-demanding cognitive tasks but are the most metabolically active at rest [40]. The most common DMN components are the MPFC, PCC/PCu and IPL. The DMN is suggested to play a role in a variety of cognitive functions, such as monitoring environmental stimuli [41], [42], reviewing past knowledge in preparation for future actions [43] and episodic memory processing [44]. To date, accumulating evidence has indicated an implication of the DMN in various brain disorders [45], [46]. In this study, we provide empirical evidence of svMCI-related abnormalities in DMN components. Interestingly, we noted that spontaneous brain activities decreased specifically in the anterior part of the DMN (i.e., the MPFC) in svMCI patients (see the following for discussions on increased spontaneous LFO in the posterior part of the DMN and the heterogeneity of svMCI-related alterations in the DMN). This decrease was in line with one previous PET study [10], which showed hypometabolism in frontal regions (including the MPFC) in svMCI patients. The MPFC is associated with executive functions, emotional regulation and social cognitive processes that are related to the self and others [47]–[49]. Thus, cognitive disturbances and neurobehavioral symptoms of svMCI patients could be attributed to the attenuated activities of the MPFC.

### Increased spontaneous LFO in svMCI

We also found several brain regions that showed increased spontaneous LFO in the svMCI patients, such as the PCC/PCu, THA and HIP. PCC/PCu are spatially located in the posterior part of the DMN. The PCC/PCu are the most metabolically active regions in healthy subjects at rest and play key roles in default mode processing [40], [41], [50], [51]. These two regions were also found to show the highest amplitudes of LFO in healthy brains [24]. In this study, we speculate that the increased spontaneous LFO in the PCC/PCu may reflect a compensation, due to vascular damage, where more resources or stronger activities are needed in svMCI patients to achieve normal levels of cognition during rest. Note that the PCC/PCu were found to show decreased LFO activities in patients with aMCI [8] and Alzheimer's disease [21], [52]. Thus, our results suggest that there may be distinct pathophysiological mechanisms between svMCI and aMCI subtypes of cognitive impairments. In addition to the PCC/PCu, the HIP also exhibited increased LFO amplitudes in the svMCI patients. HIP has been widely studied in the literature of aMCI and Alzheimer's disease [8], [21], [52], which showed functional and structural deficits in very early stage of aMCI patients and had a close relationship with memory deficits in aMCI patients. In our present study, the HIP showed increased LFO amplitudes in svMCI patients may reflect a compensatory mechanism that complements to some extent the memory deficits in the svMCI patients during rest, which may also support our hypothesis that the existence of distinct pathophysiological mechanisms between svMCI and aMCI.

### Heterogeneity of svMCI-related functional alterations in the DMN

In the current study, we found that svMCI patients exhibited decreased spontaneous LFO in the anterior part of the DMN, whereas increased spontaneous LFO were found in the posterior part compared with HC. Using R-fMRI, Sun et al. [53] found impaired functional integration between the anterior (i.e., the MPFC) and posterior (i.e., the PCC) parts of the DMN in patients with vascular cognitive impairment no dementia (VCIND), a clinical state with vascular etiology similar to that of svMCI. Growing evidence of the normal population demonstrates functional heterogeneity or segregation within the DMN [25], [54], [55]. For example, He et al. [56] found that the DMN module can be subdivided into anterior, middle and posterior subcomponents. Accordingly, the different behaviors of the DMN subcomponents may reflect unique mechanisms that are recruited by spatially distant sites of the DMN in response to vascular pathogeny of svMCI. In addtion to ALFF, we also calculated functional connectivity density and found widely decreased functional connectivity in svMCI patients. However, heterogeneity of svMCI-related functional connectivity changes were not found in the DMN. It will be interesting to investigate specific alterations within and/or between subnetworks that comprise the DMN under pathological conditions of svMCI in the future.

### Decreased GM volume in svMCI

We found widespread cortical GM volume loss in the svMCI patients that was predominantly distributed in frontal (i.e., the SFG and MFG), temporal (i.e., the STG and ITG), occipital (i.e., the MOG) and parietal (i.e., IPL) regions. The pattern of GM atrophy that was detected here was highly consistent with GM thinning in svMCI patients that has been reported in previous studies [11], [12]. Furthermore, in addition to cortical GM atrophy, we found for the first time that subcortical regions (i.e., the caudate and the thalamus) also exhibited GM loss in the svMCI patients compared to HC.

We found that large areas of the frontal lobe exhibited GM atrophy in the svMCI patients, which is consistent with a previous report of frontal cortical thinning [11]. Evidence from a meta-analysis of neurology and neuroimaging studies indicates that frontal regions are tightly related to executive functions [47], and impairments to this region can result in executive dysfunctions [57], [58]. Specifically, Seo et al. [12] found that frontal cortical thinning was associated with the executive dysfunction of svMCI patients. Therefore, our findings were consistent with previous studies. It is important to remark that previous PET studies found hypometabolism in frontal and subcortical areas of svMCI patients, and the authors argued that executive dysfunction (e.g., inertia and disinhibition) and the abnormal social behaviors of svMCI patients may be attributed to impaired fronto-subcortical circuits [10]. However, there are no reports of structural alterations in the subcortical regions of svMCI patients to date. In this study, utilizing a whole-brain VBM analysis technique, we found that the subcortical caudate and the thalamus exhibited GM atrophy in svMCI patients compared to HC. These findings provide empirical evidence that complement the hypothesis of aberrant fronto-subcortical circuits in svMCI.

Several temporal structures (i.e., the STG and ITG) also exhibited GM atrophy in the svMCI patients. Previous studies have demonstrated that the STG and the ITG show cortical thinning in patients with SVaD or svMCI [11], [59]. As a crucial node of the social perception system, the STG plays an important role in regulating complex social interactions [60]. Considering the frequently observed social behavior abnormalities and the loss of empathy in svMCI patients, our findings of the GM volume loss of the STG may account for the behavioral changes that are observed in this atypical population. Moreover, the STG and ITG are closely associated with the retrieval of auditory information [61]. Given that the svMCI patients performed worse than the HC in the AVLT recall and recognition tests, which are involved in auditory information retrieval processing, we assume that the poor behavior output (i.e., the AVLT tests) may be at least partially attributed to the structural GM atrophy in these two temporal brain regions. It should be noted that we did not find a significant correlation between temporal GM atrophy and the memory-related tests. Further large-sample work is needed to test this point.

In addition to frontal and temporal regions, the MOG and the IPL also showed GM volume loss in the svMCI patients. Occipital GM thinning has been previously reported in patients with SVaD and svMCI [11], [12]. Thus, our findings were coincident with these studies. The GM atrophy or thinning in the occipital regions may be a secondary consequence, due to the disrupted long-association fibers that are induced by vascular lesions [12], as the occipital area is the main origin and destination of these fibers [62]. Further diffusion-based studies are needed to test this hypothesis. Previous studies have suggested important implications of the IPL with respect to attention, especially in non-spatial attention processing [63]. During the task of Attention Network Test, Fernandez et al. [64] found that svMCI patients showed deficits in orienting attention networks compared with healthy controls. Thus gray matter atrophy of the IPL found in our study might provide empirical evidence to support the attention deficits mechanism of svMCI patients.

### Methodological issues

This investigation raised several issues that need to be addressed. First, the current study was cross–sectional; therefore, the longitudinal changes during the progression of the disease cannot be revealed by the data acquired here. Future follow-up studies can produce more fruitful insights into the svMCI. Second, svMCI patients exhibit different progressive trajectories, wherein some patients ultimately develop SVaD and others do not. Accordingly, full depictions of distinct patterns of brain abnormalities among different svMCI subtypes are warranted to elucidate the underlying neurophysiological mechanism that contributes to these disparate progressive trajectories and to provide unique characteristics for their identification and diagnosis.
